# Ordinary Cannulated Compression Screws or Headless Cannulated Compression Screws? A Synthetic Bone Biomechanical Research in the Internal Fixation of Vertical Femoral Neck Fracture

**DOI:** 10.1155/2018/4898301

**Published:** 2018-04-11

**Authors:** Baokun Zhang, Jingwen Liu, Wei Zhang

**Affiliations:** Shanghai Jiaotong University Affiliated Sixth People's Hospital, Shanghai, China

## Abstract

**Purpose:**

The purpose of this study is to verify whether the headless cannulated compression screw (HCCS) has higher biomechanical stability than the ordinary cannulated compression screw (OCCS) in the treatment of vertical femoral neck fractures.

**Materials and Methods:**

30 synthetic femur models were equally divided into 2 groups, with 50°, 60°, and 70° Pauwels angle of femoral neck fracture, under 3D printed guiding plates and C-arm fluoroscopic guidance. The femur molds were fixed with three parallel OCCSs as OCCS group and three parallel HCCSs as HCCS group. All specimens were tested for compressive strength and maximum load to failure with a loading rate of 2 mm/min.

**Results:**

The result showed that there was no significant difference with the compressive strength in the Pauwels angle of 50° and 60°. However, we observed that the maximum load to failure with the Pauwels angle of 50°, 60°, and 70° and the compressive strength with 70° of HCCS group showed better performance than the OCCS group.

**Conclusion:**

HCCS performs with better biomechanical stability than OCCS in the treatment of vertical femoral neck fracture, especially with the Pauwels angle of 70°.

## 1. Introduction

Femoral neck fracture in young adults is usually the result of polytrauma and high-energy injuries, for which accurate reduction and stable fixation are necessary [1, 2]. Orthopedic surgeons should choose the most effective and affordable implant to deal with this kind of fracture. Comparing with other internal fixation implants, cannulated compression screws (CCSs) have been reported for their particular superiority with the treatment of femoral neck fracture, such as less damage of soft tissue, less blood loss, and being easy to operate, making them become one of the most common fixation devices [3]. However, with the increase of Pauwels angle, high rates of fixation failure while fixing with CCS, including femoral neck shortening, loose fixation, varus deformity, and fracture displacement, have been reported.

Several factors might affect the stability between fracture fragments while using the CCS in treating femoral neck fracture such as the direction of the screws, the number of the screws, the position of the screws, the configuration of the screws, and the type of the screws. It has been proved that there is no difference in compressive strength among screws placement angle [4]. There are many researches trying to figure out the best choice of the number of screws but it still remains controversial. Several studies have shown evidence about the relationship between the position of cannulated screw and the effect [5–7], and the triangular or inverted triangular configuration performed with better strength and stability of fixation [8]. Then does the type of CCS have effect on the stability of femoral neck fracture? There are several literatures that can be searched but only one literature focused on the length of thread, and no biomechanical research was conducted [9]. HCCS has been introduced for the treatment of femoral neck fractures in recent years, functions of which are via whole thread and continuous compression with proximal lateral femoral cortex to femoral head.

The purpose of this study is to verify the assumption that the HCCS has better biomechanical stability.

## 2. Materials and Methods

### 2.1. Specimen Preparation

**Figure 1:**
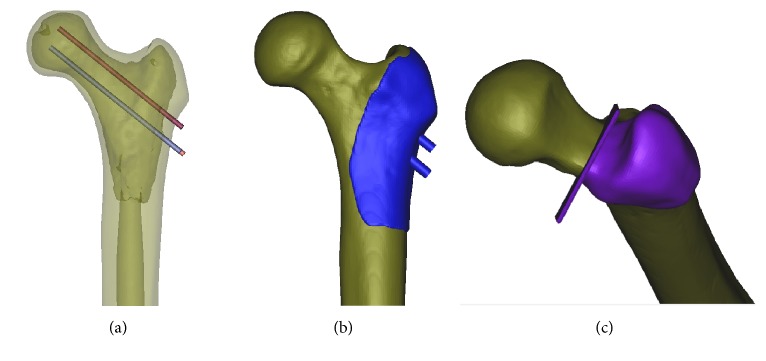
The design of guiding plates made with 3D printing technology.

**Figure 2:**
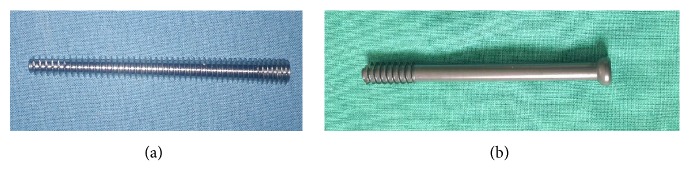
*The two types of compression cannulated screw*. (a) Headless compression cannulated screw. (b) Ordinary compression cannulated screw.

30 same shaped left side synthetic femur models (ENOVO, China) were equally divided into two groups, and each group was further equally divided by three different Pauwels angles (50°, 60°, and 70°). To ensure the site of each screw at exact the same position, the first screw was driven up to the subchondral bone just beneath articular side of the femoral head; the second screw was placed beneath the first screw near to anterior cortex and the third one was placed near to posterior cortex; three screws form the configuration of standard triangle. We designed a guiding plate and made it with 3D printing technology. Fracture line was made with a medical pendulum saw just from the upper side of the lesser trochanter proximal to the superior femoral neck with the assistance of 3D printing guide plates, to avoid the appearance operation error (Figure 1). Firstly, under the C-arm fluoroscopic guidance and guiding plate, 3 parallel guide pins were placed into molds first. Then we removed the guiding plate and predrilled 3 insertion holes with 3.0 mm width drill bit through the guide pin, and after the accurate reduction of fracture, three 6.5 mm cannulated screws were inserted. Finally, the fracture was repaired with three CCSs. Group A (OCCS group): three parallel OCCSs (Stryker Co.) of 6.5 mm width placed with the configuration of triangle; Group B (CCCS group): three parallel HCCSs (Acumed Co.) placed with the configuration of triangle (Figure 2).

### 2.2. Biomechanical Testing

**Figure 3:**
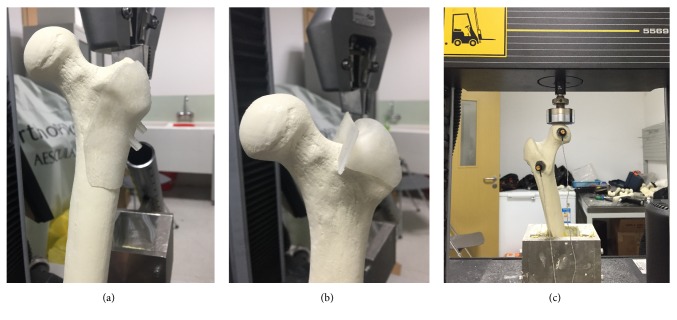
*The preparation of the models*. (a, b) The guiding plates were used to obtain the accurate angle of fracture line and ideal placement of screws. (c) Two magnets were placed on the proximal and distal fragments, respectively, to record the displacement of the two fragments.

All tests were performed with axial compressive loading with an Instron test system (Instron, Norwood, MA, USA) which included a base, a pressure applicator, and a data analyzer. The distal femur was fixed with shaft adduction angle of 7° using dental powder to imitate the femur form in normal walking. A vertical force was put on the top of the femoral head at the loading rate of 2 mm/min. The failure load was defined as the marked decrease followed by the maximum load or the fragments displaced with 5 mm [10]. Two magnets were placed on the proximal and distal fragments, respectively, to record the displacement of the two fragments (Figure 3).

### 2.3. Statistical Analysis

The analysis was performed with the use of SPSS software (SPSS Version 20; SPSS Inc., Chicago, IL, USA). To detect the differences of compressive strength and failure load, the two groups were performed using *t*-test with significance set at *P* < 0.05.

## 3. Results

**Figure 4:**
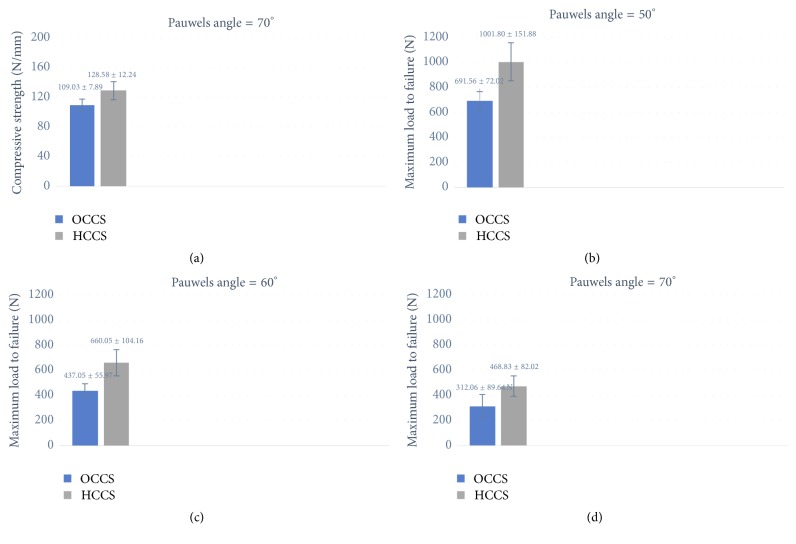
*The results of the biomechanical study with statistical significance*. (a) The compressive strength with Pauwels angle of 70°. (b, c, d) The maximum load to failure with Pauwels angle of 50°, 60°, and 70°.

The testing results of compressive strength are showed in Table 1. We found that in our vertical fracture models the compressive strength of HCCS group performed better than the OCCS group with the Pauwels angle of 70° (109.03 ± 7.89 versus 128.58 ± 12.24, *P* = 0.019), but there is no statistical significance between two groups with 50° (177.58 ± 25.74 versus 214.08 ± 18.62, *P* = 0.133) and 60° (137.54 ± 32.57 versus 135.96 ± 43.52, *P* = 0.721). And the maximum failure load of the two groups is performed in Table 2. The results clearly indicate that the maximum load to failure in the HCCS group performed significantly better than that in OCCS group (Figure 4).

## 4. Discussion

The incidence of femoral neck fracture has increased rapidly in recent years because of the acceleration of ageing with the population process. Different from the old, most of the young patients suffered from high-energy injury directly to the femoral neck, for whom choosing an appropriate device is significant. In recent years, the use of new methods to treat femoral neck fracture has been reported a lot. For example, the study of Samsami et al. [11] on vertical femoral neck fracture of young people compared the CCS with proximal femoral locking plate (PFLP) and the combination of DHS and an antirotation screw. The result revealed that the latter had better resistance of rotation shear force. Further, a new type of femoral neck locking plate (FNLP) consisting of a locking plate and five screws showed satisfactory biomechanical results in Pauwels type III femoral neck fracture. Compared with DHS, the new type of FNLP performed better in the biomechanical stability and it also could reduce the incidence of bone nonunion effectively by conduct force to the femoral neck dispersedly via the multiaperture screw system [10]. In Zhu et al. [12] follow-up study including 74 patients with femoral neck fracture, they introduced a new treatment for femoral neck fracture with percutaneous compression plate. The results showed that 98.5% of patients had good prognosis and could walk unaided again. Only two of them appeared with avascular necrosis of femoral head and delayed union, respectively. Although there are so many choices for treatment, none of them has been proven to be overwhelmingly superior.

The use of OCCS in the treatment of femoral neck fracture achieved great success [13]. A 17-item survey showed that for the undisplaced fracture near 80% surgeons prefer using OCCS [14]. In the retrospective study of 59 patients in a single institution, 4/5 of them treated with OCCS showed good results at last [15]. However, for the more vertical fracture, the rate of failure and complication has risen obviously because of the increase of shear force [16, 17]. In our study, the compressive strength of OCCS of the Pauwels angle of 50°, 60°, and 70° is 177.58 ± 25.74 N/mm, 137.54 ± 32.57 N/mm, and 109.03 ± 7.89 N/mm, respectively. And the maximum load to failure is 691.56 ± 72.02 N, 437.05 ± 55.97, and 312.06 ± 89.64 N. It appeared that with the increase of Pauwels angle the axial stiffness and the maximum load to failure decrease obviously.

The HCCS has been introduced as a reliable choice of internal fixation in recent years. The diameter of HCCS becomes larger from screw tip to tail, and the screw pitch becomes smaller to make the screw tip goes faster than the tail while going into the bone, and it forms compression between fractures. The design of the screw thread increases the contact between the screw and the bone, and the conical shape makes it possible to obtain greater holding force, pull-out strength, and shear strength. Thereby it can increase the compression in joint and create an immediate stability to help early mobilization. Dodds et al. [18] found that long thread screws provided optimal fixation when used for the scaphoid fracture. In the retrospective study with 41 distal ulna patients with rheumatoid arthritis, the patients with the treatment of the HCCS showed better stability and higher rate of bone union, compared to those treated with OCCS [19]. As for the treatment of metacarpal neck fractures, the HCCS also shows its advantages in earlier mobilization than those fixed with K-wires [20]. Moreover, in the case reported by Borse et al., they introduced a method using two HCCS in the treatment of Hoffa fracture and have achieved satisfying result [21]. And the method gained affirmation and improvement in the prospective study of Li et al. [22]; eight Chinese patients suffering isolated Hoffa fractures were treated with HCCS combined with back buttress plate; the result is inspiring with all fractures healed clinically and the range of motion of the knee joint became better. With all the successful use, the HCCS has become an effective internal fixation device. In addition, the compressive strength with the Pauwels angle of 70° (128.58 ± 12.24 N/mm) also showed satisfactory results in our biomechanical study. And the load of failure showed good results as well; with 50°, 60°, and 70° Pauwels angle, the loads were 1001.80 ± 151.88 N, 660.05 ± 104.16 N, and 468.83 ± 82.02 N, respectively.

We strongly proved the advantages of HCCS in treatment of the vertical femoral neck fracture. Especially with the Pauwels angle of 70°, no matter the axial stiffness (128.58 ± 12.24 versus 109.03 ± 7.89, *P* = 0.019) or the maximum load to failure (468.83 ± 82.02 N versus 312.06 ± 89.64 N, *P* = 0.018), the superiority of biomechanical stability of HCCS is obvious.

However, there are several limitations for this experiment. Firstly, the bone is synthetic femur model rather than cadaveric femur bone. Secondly, limited to implants supply, we have failed to consider the length of thread purchase and screw arm of the OCCS, which might affect the results.

## 5. Conclusion

In summary, with the vertical femoral neck fracture especially Pauwels angle of 70°, HCCS performs with much better biomechanical stability than OCCS. And HCCS can be introduced a better implant than OCCS while treating the vertical femoral neck fracture. However, further investigation with clinical research is needed in the future.

## Figures and Tables

**Table 1:** The results of compressive strength in two groups.

Pauwels angle	50	60	70
Compressive strength (N)			

Group A (OCCS)			
Mean	177.58	137.54	109.03
Minimum	135.00	114.43	91.14
Maximum	195.30	193.84	120.35
SD	25.74	32.57	7.89
Group B (HCCS)			
Mean	214.08	135.96	128.58
Minimum	192.09	77.89	113.40
Maximum	240.47	199.69	153.21
SD	18.62	43.52	12.24
*P*	0.133	0.721	0.019

**Table 2:** The results of maximum load to failure in two groups.

Pauwels angle	50	60	70
Maximum failure load (N)			

Group A (OCCS)			
Mean	691.56	437.05	312.06
Minimum	567.81	391.27	246.02
Maximum	750.39	530.00	467.81
SD	72.02	55.97	89.64
Group B (HCCS)			
Mean	1001.80	660.05	468.83
Minimum	809.77	508.98	324.34
Maximum	1202.56	772.21	521.18
SD	151.88	104.16	82.02
*P*	0.022	0.026	0.018
